# Assessment of Condyle and Glenoid Fossa Morphology Using CBCT in South-East Asians

**DOI:** 10.1371/journal.pone.0121682

**Published:** 2015-03-24

**Authors:** May Al-koshab, Phrabhakaran Nambiar, Jacob John

**Affiliations:** Department of Diagnostic and Integrated Dental Practice, Faculty of Dentistry, University of Malaya, Kuala Lumpur, Malaysia; University of Palermo, ITALY

## Abstract

**Introduction:**

Proper imaging allows practitioners to evaluate an asymptomatic tempormandibular joint (TMJ) for potential degenerative changes prior to surgical and orthodontic treatment. The recently developed cone-beam computed tomography (CBCT) allows measurement of TMJ bony structures with high accuracy. A study was undertaken to determine the morphology, and its variations, of the mandibular condyle and glenoid fossa among Malay and Chinese Malaysians.

**Methods:**

CBCT was used to assess 200 joints in 100 subjects (mean age, 30.5 years). i-CAT CBCT software and The Mimics 16.0 software were employed to measure the volume, metrical size, position of each condyle sample and the thickness of the roof of the glenoid fossa (RGF).

**Results:**

No significant gender differences were noted in thickness of the RGF and condylar length; however condylar volume, width, height and the joint spaces were significantly greater among males. With regards to comparison of both TMJs, the means of condylar volume, width and length of the right TMJ were significantly higher, while the means of the left condylar height and thickness of RGF were higher. When comparing the condylar measurements and the thickness of RGF between the two ethnic groups, we found no significant difference for all measurements with exception of condylar height, which is higher among Chinese.

**Conclusion:**

The similarity in measurements for Malays and Chinese may be due to their common origin. This information can be clinically useful in establishing the diagnostic criteria for condylar volume, metrical size, and position in the Malaysian East Asians population.

## Introduction

The tempormandibular joint (TMJ) is one of the most important joints in the body, and it has a close relationship with the oral cavity and teeth. The position and function of the mandibular condylar portion of the TMJ is directly controlled by the oral structures, including the associated muscles. Therefore, treatment performed by orthodontists can influence TMJs. However, the presence of TMJ abnormalities and symptoms play a critical role in orthodontic treatment planning and are important to evaluate prior to commencing treatment [1]. As the primary center of growth in the mandible, the condyle responds to continuous stimuli throughout the remodeling process, and thus plays an important role in the final dimensions of the adult mandible. Its volume and size can be related to the final dimensions of the mandibular as well as to the final relationship between maxillary and mandibular arches. Examination of TMJ structures radiographically is very important for evaluating the abnormalities and bony changes that affect the TMJ [2]. Several studies have reported high accuracy when using cone-beam computed tomography (CBCT (to evaluate the TMJ region. According to Honda et al. (2004)^,^ their CBCT results showed no statistically significant differences from the actual measurements, although the measurements were done in micrometers. The purpose of this study was to determine the morphology of the condyle and glenoid fossa among a selected population with normal TMJ to recognize variations.

## Materials and Methods

**Fig 1:**
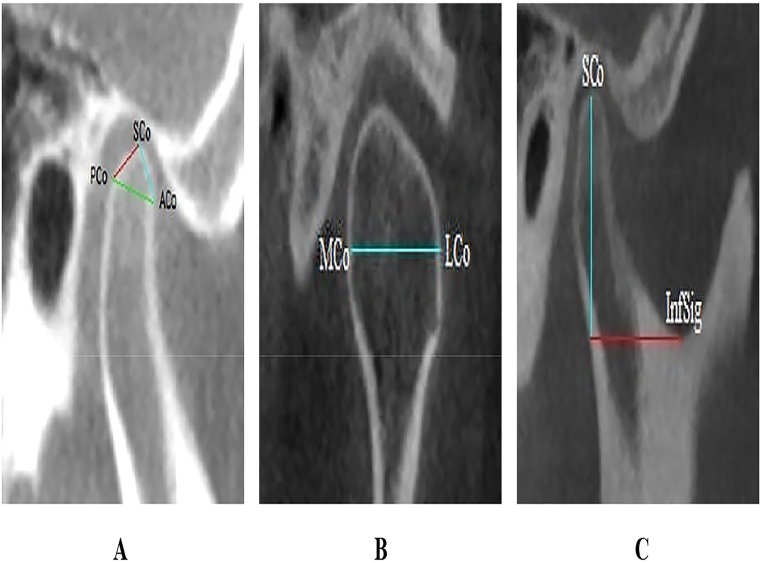
Condyle size measurements. CBCT images show the methods of measuring the condyle size. **1A. Sagittal view showing condylar length measurement. 1B. Coronal view showing condylar width measurement. Fig. 1C. Sagittal view showing condylar height measurement.**

This study received the Faculty Ethical Committee’s approval prior to commencement (IRB approval no. DF DP1408/0068[P]). The committee was aware that this was a retrospective study and that it was undertaken using patients’ data and radiographs. As this is a teaching institution, all patients seeking treatment from the Faculty of Dentistry are informed of the possibility that all forms of their records may be used for teaching and research purposes, and verbal consent is taken with the assurance that their identity will remain anonymous. CBCT images were obtained with the i-CAT Imaging System (Imaging Sciences International Inc. Hatfield, USA). All images were taken following a standardized protocol for patient positioning and exposure parameter setting (120kVp, 3–7mA, 20 sec) and image acquisition at 0.3mm voxel size. The sample comprised 100 CBCT images of patients of Malay and Chinese ethnicity who visited the Oral and Maxillofacial Imaging Division of the faculty. The sampling was random with the purposive requirement of there being equal numbers according to gender and ethnicity to avoid any possible effect for these factors. The patients were aged between 18–45 years with an average age of 30.5 years. For each patient, both the right and left side images were taken, therefore the total number of TMJ images studied was 200. Images that showed any pathology in the condyle or the glenoid fossa, fractures including those of the mandible that affected the condyle or its position, poor image quality and loss of patient maximum intercuspation were excluded. The study was designed to analyze metrically the morphology of the condyle and glenoid fossa, which included the condylar size (length, width and height), thickness of the glenoid fossa roof, position of the condyle and condylar volume. Linear measurements of the TMJ were carried out using i-CAT classic software and the condylar volume was recorded using MIMICS 16.0 software (Materialise, NV, Belgium). The methodology used in this study to measure the size of the condyle was described by Hilgers [3]. A two-dimensional sagittal slice was selected in which the condyle and glenoid fossa were clearly noticed. From this slice the condylar length was measured. The condylar length was measured from the line extending from the posterior mandibular condyle point (PCo) to the anterior mandibular condyle point (ACo). Both these points are located 4 mm inferior to the superior mandible condyle (SCo) on either side of the condyle (Fig. 1A). Condylar width, which is the linear distance between the medial and lateral mandible poles, was measured in the coronal plane (Fig. 1B). Condylar height was measured as a perpendicular linear distance from superior mandible condyle (SCo) to a line constructed between the most inferior point of the sigmoid notch (InfSig) perpendicular to the tangent of the posterior surface of the ramus in the sagittal plane (Fig. 1C).

**Fig 2:**
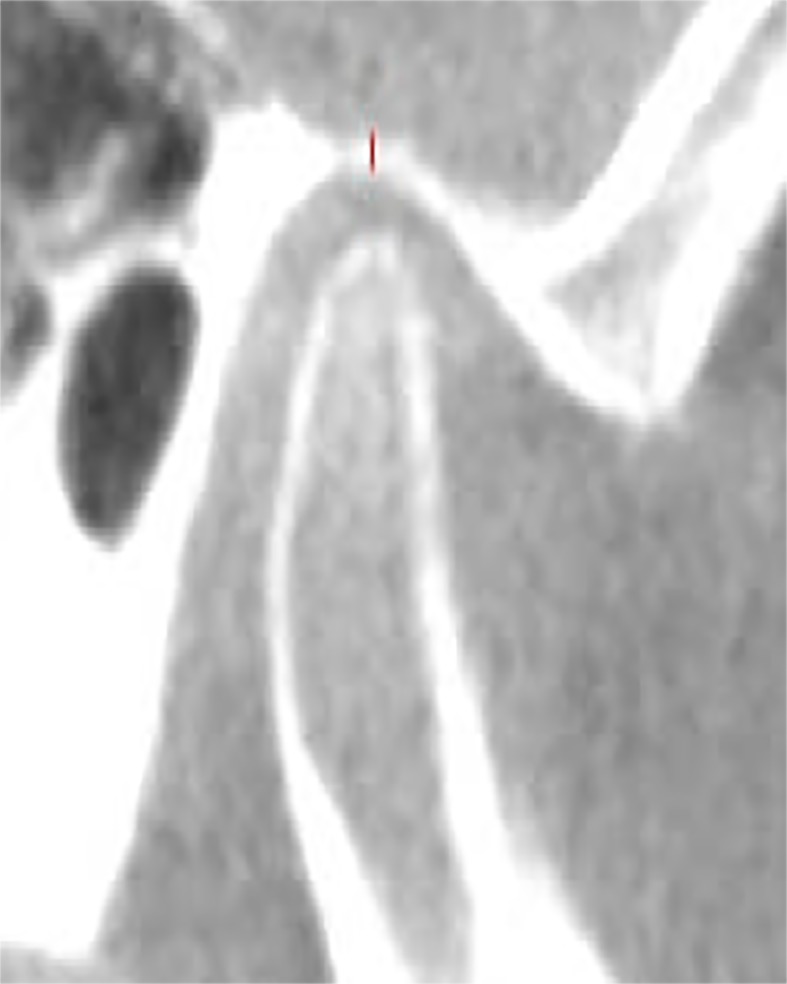
Sagittal view showing genoid fossa roof thickness measurement.

**Fig 3:**
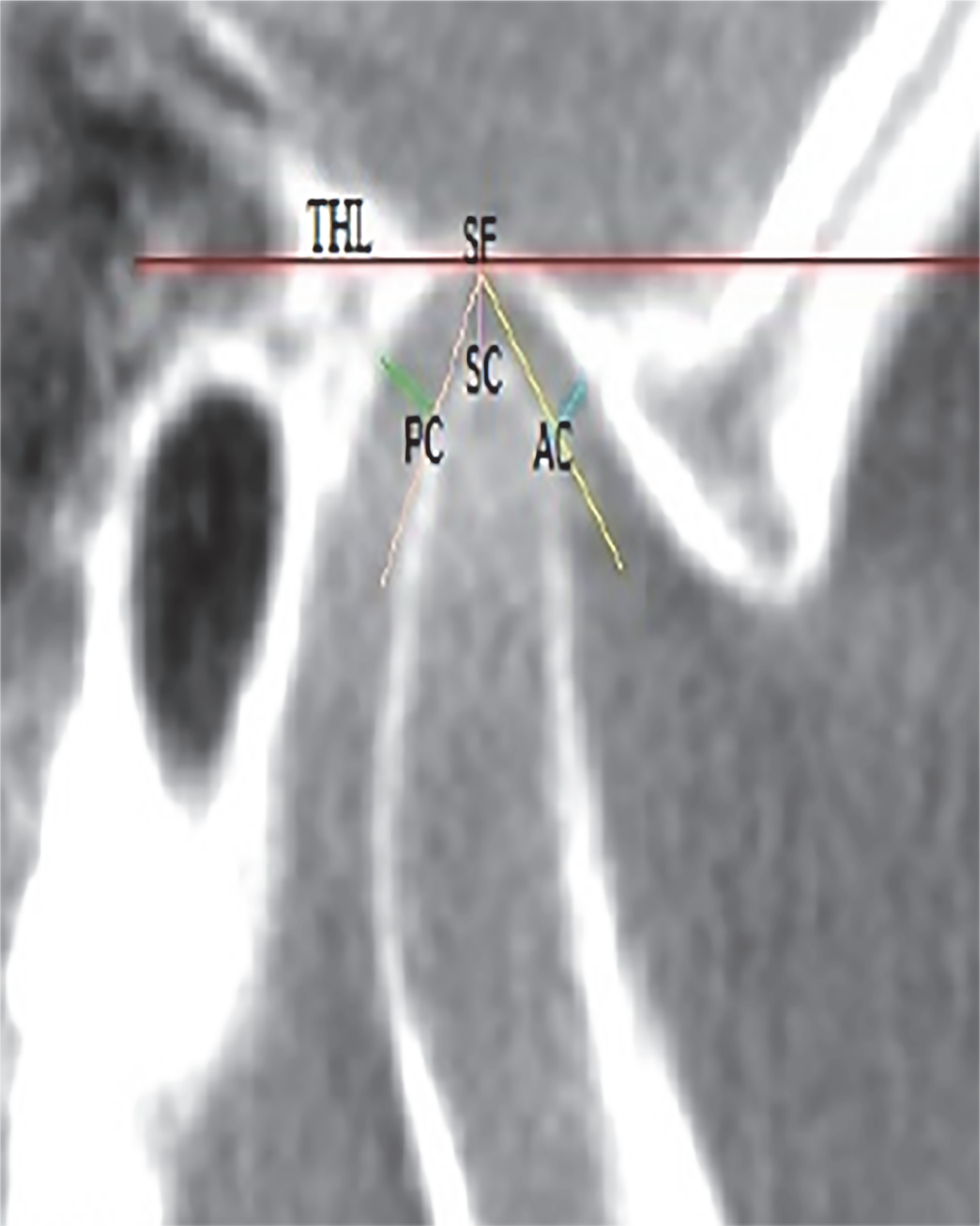
Sagittal view showing condyle joint space measurements.

The thinnest bone forming the roof of the glenoid fossa (RGF) was identified and measured in the sagittal plane [4], (Fig. 2). The position of the condyle was determined by measuring of joint spaces. The landmarks and linear measurements of the space between the condyle and the glenoid fossa were determined [5]. The true horizontal line (THL) which is tangential to the roof of the glenoid fossa was used as the reference plane. The superior space (SS) was measured as a distance from the superior condyle (SCo) (most superior condyle point) to the superior fossa (SF). In order to measure the anterior and posterior spaces, the line tangent to the most prominent anterior and posterior aspects of the condyle was drawn from the SF. The distance from the anterior condyle (AC) to the corresponding glenoid fossa bone was measured as the anterior space (AS) and from the posterior condyle (PC) to the corresponding glenoid fossa bone was measured as the posterior space (PS) (Fig. 3).

**Fig 4:**
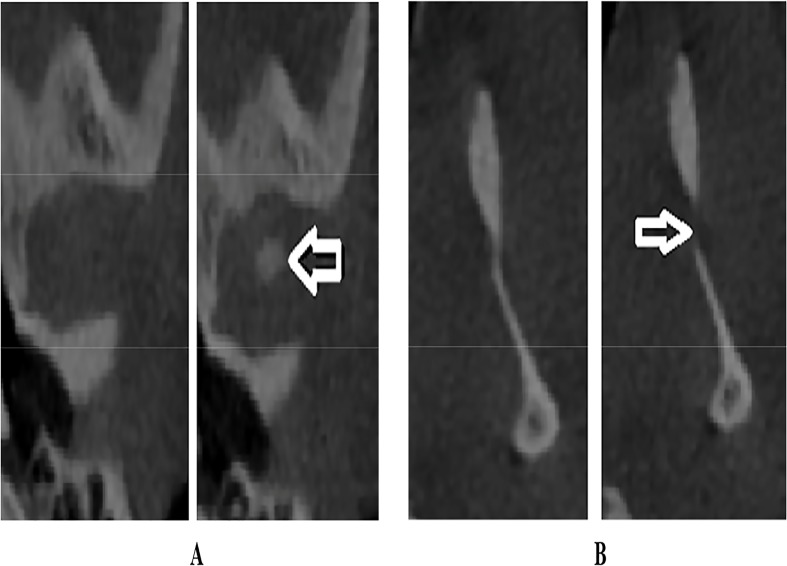
A. Axial view showing superior limit of the condyle. B. Axial view showing inferior limit of the condyle.

**Fig 5:**
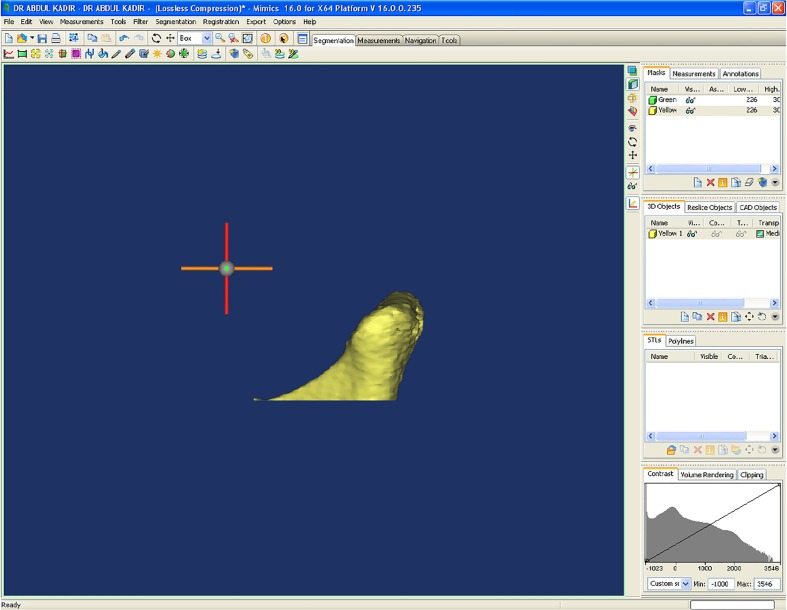
3D Condyle reconstruction using Mimics software.

Mimics 16.0 software (Materialise, NV, Belgium) was used to measure the volume of each condyle. On the axial view, the superior limit of the condylar head was determined when the first radiopaque point appeared in the joint space while scrolling the axial images from the upper to the lower regions of the joint space (Fig. 4-A); the inferior limit was determined when the sigmoid notch, which is between the mandibular condyle and the coronoid process disappeared (Fig. 4-B). Then, separation of the structures around the mandibular condyle was done. After the condylar segmentation, 3-D reconstructions were produced (Fig. 5) and volumetric assessment (mm^3^) was made for each condyle through the Mimics automatic function. To assess the significance of any errors during measurement, 10% of all samples were re-evaluated randomly thrice within a one week interval. Intra-class correlation coefficients of 0.98 were achieved. Therefore, reproducibility of the evaluation method was acceptable.

## Results

The measurements were processed and analyzed using SPSS 20.0. For all variables, the mean and standard deviation were calculated, based on gender, ethnicity, and TMJ sides (Table 1). The distribution of quantitative variables (condylar size, condylar volume, thickness of RGF, AS, PS, and SS) was examined for normality using the Shapiro-Wilk test before analysis. The Independent t-test or Mann Whitney test was used where appropriate to examine the difference in mean between gender (male and female) and ethnicity (Malay and Chinese). The Paired t-test or Wilcoxon test was used where appropriate to examine the difference between the right and left sides.

**Table 1:** Statistical data for the subjects.

	Mean(SD) of the variables(***mm***)					
	TMJ side		Gender		Ethnicity	
	Left	Right	Male	Female	Malay	Chinese
**RGF thickness**	1.24(0.90)	1.00(0.87)	1.20(0.90)	1.14(0.78)	1.20(0.90)	1.00(0.70)
**Anterior space**	1.68(0.60)	1.79(0.70)	1.78(0.70)	1.50(0.77)	1.68(0.57)	1.79(0.73)
**Superior space**	2.70(1.50)	3.00(1.50)	3.30(1.20)	2.40(1.20)	2.90(0.95)	3.09(1.42)
**Posterior space**	1.96(1.06)	2.14(1.20)	2.16(1.50)	1.90(0.88)	2.14(1.05)	2.00(1.19)
**Condylar length**	7.08(1.02)	7.31(1.01)	7.29(1.01)	7.11(1.03)	7.50(1.20)	7.20(1.77)
**Condylar width**	17.17(2.45)	17.27(2.43)	17.93(2.46)	17.04(2.35)	17.18(2.45)	17.80(2.41)
**Condylar height**	17.88(3.25)	17.49(2.98)	18.25(3.18)	17,22(3.25)	17.00(3.16)	18.37(2.94)
**Condylar volume** *	1450.89 (609.93)	1460.69 (609.46)	1613.87 (725.57)	1339.65 (494.93)	1452.99 (751.73)	1459.86 (524.12)

*in *mm* ^*3*^

The results in Table 2 show that there was no significant difference in the RGF thickness between genders. However, there was a significant difference between genders when anterior joint space (AS), superior joint space (SS), and posterior joint space (PS) were compared. The ratios of SS and PS to AS, with AS set to 1.0, were 1.7 and 1.3, respectively. There was no significant difference between males and females when condylar length was compared. In addition, the mean values of condylar width, height, and volume in males were higher compared to females. Comparison was done between ethnic groups to determine any variation amongst them. The results showed no significant differences between Malays and Chinese in most of the measurements, (Table 3) However, condylar height in the Chinese was the only feature that was larger than in the Malays.

**Table 2:** Comparison the dimensions of the condyle and glenoid fossa between males and females.

Dependent variable	Males, n = 100, Mean (SD)***mm***	Females, n = 100, Mean (SD)***mm***	Mean diff. (95% CI)	P value
RGF thickness	1.20(0.90)^c^	1.14(0.78)^c^	———-	0.097^b^
Anterior space	1.78(0.70)^c^	1.50(0.77)^c^	———-	0.006^b^
Superior space	3.30(1.20)^c^	2.40(1.20)^c^	———-	0.000^b^
Posterior space	2.16(1.50)^c^	1.90(0.88)^c^	———	0.006^b^
Condylar length	7.29(1.01)	7.11(1.03)	0.17(-0.10,0.46)	0.223^a^
Condylar width	17.93(2.46)	17.04(2.35)	0.89(0.22,1.56)	0.009^a^
Condylar height	18.25(3.18)	17,22(3.25)	-1.03(0.13,1.93)	0.036^a^
Condylar volume*	1613.87(725.57)^c^	1339.65(494.93)^c^	———-	0.000^b^

a. Independent t-test;

b. Mann-Whitney test;

c. Median (IQR). Level of significant was set at 0.05;

* in *mm* ^*3*^.

**Table 3:** Comparison of the dimensions of the condyle and glenoid fossa between the Malays and Chinese.

Dependent variable	Malays, n = 100, Mean (SD)***mm***	Chinese, n = 100, Mean (SD)***mm***	Mean diff. (95% CI)	P value
RGF thickness	1.20(0.90)^c^	1.00(0.70)^c^	————	0.096^b^
Anterior space	1.68(0.57)	1.79(0.73)	-0.11(-0.29,0.07)	0.233^a^
Superior space	2.90(0.95)	3.09(1.42)	-0.18(-0.52,0.15)	0.276^a^
Posterior space	2.14(1.05)^c^	2.00(1.19)^c^	————	0.842^b^
Condylar length	7.50(1.20)^c^	7.20(1.77)^c^	———-	0.372^b^
Condylar width	17.18(2.45)	17.80(2.41)	-0.61(-1.29,0.05)	0.074^a^
Condylar height	17.00(3.16)	18.37(2.94)	-1.36(-2.21,0.51)	0.002^a^
Condylar volume*	1452.99(751.73)^c^	1459.86(524.12)^c^	———	0.277^b^

a. Independent t-test;

b. Mann-Whitney test;

c. Median (IQR); Level of significant was set at 0.05;

* in *mm* ^*3*^.

According to the data that are shown in Table 4, the anterior, superior, and posterior joint spaces show no significant difference between the right and left sides. On the other hand, the mean condylar volume, length, and width of the right TMJ were significantly higher than the left, whereas, the mean condylar height, and thickness of RGF on the left side were significantly higher than on the right side.

**Table 4:** Comparison of the dimensions of the condyle and glenoid fossa structures between Left and Right sides.

Dependent variable	Left, n = 100, Mean (SD)***mm***	Right, n = 100, Mean (SD)***mm***	Mean diff. (95% CI)	P value
RGF thickness	1.24(0.90)^c^	1.00 (0.87)^c^	—————	0.012^b^
Anterior space	1.68(0.60)	1.79(0.70)	-0.10 (-0.27,0.05)	0.181^a^
Superior space	2.70(1.50)^c^	3.00(1.50)^c^	—————	0.439^a^
Posterior space	1.96(1.06)^c^	2.14(1.20)^c^	————-	0.211^a^
Condylar length	7.08(1.02)	7.31(1.01)	-0.23(-0.41,0.04)	0.015^a^
Condylar width	17.17(2.45)	17.27(2.43)	0.43(0.08,0.79)	0.016^a^
Condylar height	17.88(3.25)	17.49(2.98)	0.53(0.01,1.05)	0.043^a^
Condylar volume*	1450.89(609.93)^c^	1460.69(609.46)^c^	———-	0.048^a^

a. Paired t-test;

b. Wilcoxon test;

c. Median (IQR); Level of significant was set at 0.05;

* in *mm* ^*3*^.

## Discussion

The morphology of the mandibular condyle and glenoid fossa varies greatly according to age group and gender [6], therefore it is necessary to recognize variations or abnormalities in these structures, especially when performing orthodontic management and orthognanthic surgery [1]. In this study, we excluded older subjects because they may have had the progressive degenerative bone changes that cause TMJ osteoarthritis [7].

The relationship between gender and RGF thickness was discussed by Honda [8], Ejima [9], and Kijima [10], all of them agreed that there was no significant difference in RGF thickness between males and females, and this was also confirmed by this study. In our study, the value of SS was the greatest in both sexes, followed by PS and AS respectively. This result is in agreement with the results of Ikeda & Kawamura [5], Dalili [11], and Kinniburgh [12]. Kinniburgh’s [12] and Dalili’s [11] results agree with the results of our study that males have larger joint spaces than females especially the SS and PS. These larger joint spaces in males could possibly be explained by a greater soft tissue thickness. Condylar volume, width and height in males are larger than in females. Tadej [13] reported that overall size of the condyle in males was significantly larger than in females, probably due to the difference in overall size of the condyle between males and females in general.

In this study, there was a significant difference between sides when comparing the thickness of the glenoid fossa. This asymmetry could be related to normal cranial base asymmetries. The relationship between sides (right and left) and joint spaces (AS, SS and PS) was discussed by Wang [14] and Rodrigues [15]. They agreed that there was no significant difference between left and right sides and this was also confirmed by this study. Marmary [16] hypothesized that a midline drawn between the foramina spinosa would be a reliable reference for submentovertex (SMV) projection. Measurements were made from the skull structures to this constructed midline. Their results showed craniofacial bones to be quite symmetrical in relation to the center line as the deviation was within a millimeter. They regarded this amount of asymmetry as 'normal asymmetry.' Cohlmia [17] suggested that the position of the condyle is asymmetric in a normal population. Blaschke & Blaschke [18] found that there was a variation in condyle position in normal joints. In this study, we might assume that this result represents normal people and those with malocclusions. Condylar volume, length, and width were significantly larger on the right side while condylar height and thickness of the roof of the glenoid fossa were significantly larger on the left side. This is could be explained by the present of different types of malocclusions in our sample. The condyle asymmetry between the right and left sides observed in the presence study could possibly be explained by the presence of a preferred side for mastication in subjects with malocclusion [17].

There was no study in the literature that discussed variations in condyle and glenoid fossa morphology within the same ethnicity and between ethnicity. In general, there was no significant difference between Malays and Chinese except when comparing the condylar height. The similarity in the condylar and glenoid fossa measurements for Malays and Chinese may be due to their common East Asians ancestry.

The articular disc cannot be evaluated by CBCT, so the information obtained from this study depends upon analysis of the bony structures of the TMJ viewed from CBCT images only.

Future prospective studies are required to assess the TMJ using both CBCT and MRI to evaluate the TMJ bony structures and the articular disc.

## Conclusion

This study clearly showed variations in condyle dimensions and glenoid fossa roof thickness. This anatomical variability in the dimensions may be clinically important during surgical procedures such as orthognanthic surgery and also when performing orthodontic management. The position of the condyle, thickness of RGF and the condyle size can be an indicator for various TMJ joint diseases such as discectomy, disc perforation, disc displacement and degenerative joint disease. In general, males exhibited a larger condyle volume and size than females. In the assessment of symmetries between the condyles, they are asymmetrical and therefore each condyle must be evaluated separately. This information can be clinically useful in establishing the diagnostic criteria for condylar volume, size and position among the Malaysian East Asians population. A careful assessment of this area during pre-operative planning procedures is important. CBCT cross-sectional imaging may serve this purpose.

### Ethical approval

This project was approved by the Faculty of Dentistry, University of Malaya Medical Ethics Committee (IRB approval no. DF DP1408/0068[P]).

## Supporting Information

S1 Table Mandibular condyle and glenoid fossa measurements. (DOCX)

S2 Table Measurements for the reliability test. (DOCX)
