# Supplementary material for: Assessment of Condyle and Glenoid Fossa Morphology Using CBCT in South-East Asians
Source: PLoS One. 2015 Mar 24;10(3):e0121682. doi: 10.1371/journal.pone.0121682 (PMC4372412; doi:10.1371/journal.pone.0121682)
Supplement: S1 Table — (DOCX) [file pone.0121682.s001.docx]

**Supporting Information**

**(S1)**

**S (1)**

**Table 1. Measurements of the thickness of the glenoid fossa roof, anterior joint space, superior joint space, posterior joint space, condylar length, condylar width, condylar height and condylar volume.**

| **No** | **Gender** | | **Age** | | **Race** | | **Side** | **Glenoid Roof Thickness** | | **Condylar Position** | | | | | | **Condylar Length** | | **Condylar Width** | | **Condylar**  **height** | | **Condylar volume** | |
| --- | --- | --- | --- | --- | --- | --- | --- | --- | --- | --- | --- | --- | --- | --- | --- | --- | --- | --- | --- | --- | --- | --- | --- |
|  |  |  |  |  |  |  |  |  |  | **AS** | | **SS** | | **PS** | |  |  |  |  |  |  |  |  |
| 1 | F | | 19 | | Chinese | | Left | 2.01 | | 2.42 | | 6.75 | | 6.00 | | 6.52 | | 18.62 | | 18.30 | | 1255.00 | |
|  |  |  |  |  |  |  | Right | 1.70 | | 2.50 | | 5.51 | | 5.90 | | 6.63 | | 18.91 | | 17.70 | | 1821.36 | |
| 2 | F | | 23 | | Chinese | | Left | 1.60 | | 1.50 | | 2.10 | | 2.66 | | 6.70 | | 21.60 | | 17.60 | | 1182.85 | |
|  |  |  |  |  |  |  | Right | 1.24 | | 2.40 | | 2.00 | | 2.50 | | 5.50 | | 22.80 | | 17.86 | | 1248.62 | |
| 3 | F | | 33 | | Chinese | | Left | .76 | | 2.01 | | 2.10 | | 2.16 | | 5.70 | | 22.40 | | 15.30 | | 1322.69 | |
|  |  |  |  |  |  |  | Right | .90 | | 1.90 | | 2.10 | | 2.16 | | 5.70 | | 22.50 | | 15.02 | | 1035.53 | |
| 4 | F | | 26 | | Chinese | | Left | .88 | | 1.40 | | 1.50 | | 1.50 | | 5.20 | | 19.20 | | 15.00 | | 1192.60 | |
|  |  |  |  |  |  |  | Right | .60 | | .60 | | 2.10 | | 1.88 | | 6.93 | | 18.30 | | 13.20 | | 1250.71 | |
| 5 | F | | 41 | | Chinese | | Left | .90 | | 1.70 | | 2.10 | | 1.70 | | 5.88 | | 15.90 | | 15.00 | | 1751.49 | |
|  |  |  |  |  |  |  | Right | .70 | | 1.60 | | 2.10 | | 1.50 | | 6.00 | | 15.91 | | 14.70 | | 1875.80 | |
| 6 | F | | 40 | | Chinese | | Left | .90 | | 2.40 | | 1.80 | | 1.72 | | 7.80 | | 21.60 | | 14.40 | | 1069.35 | |
|  |  |  |  |  |  |  | Right | .90 | | .85 | | 2.40 | | 1.70 | | 8.40 | | 18.30 | | 15.00 | | 1236.63 | |
| 7 | F | | 31 | | Chinese | | Left | .90 | | 1.50 | | 1.20 | | .50 | | 7.20 | | 11.10 | | 23.40 | | 1007.34 | |
|  |  |  |  |  |  |  | Right | 1.50 | | 2.16 | | 1.20 | | 1.08 | | 6.90 | | 12.30 | | 21.10 | | 1412.89 | |
| 8 | F | | 25 | | Chinese | | Left | .60 | | .95 | | .90 | | 1.50 | | 6.60 | | 13.20 | | 16.80 | | 1701.57 | |
|  |  |  |  |  |  |  | Right | .60 | | 1.20 | | 1.27 | | 1.27 | | 7.20 | | 15.00 | | 17.10 | | 1585.16 | |
| 9 | F | | 37 | | Chinese | | Left | .90 | | 1.70 | | 2.10 | | 1.53 | | 7.80 | | 18.90 | | 16.80 | | 1449.25 | |
|  |  |  |  |  |  |  | Right | .60 | | 1.20 | | 2.10 | | 1.24 | | 8.10 | | 18.30 | | 16.50 | | 1440.67 | |
| 10 | F | | 32 | | Chinese | | Left | .67 | | 1.08 | | 1.34 | | 1.08 | | 7.13 | | 15.60 | | 18.60 | | 1320.92 | |
|  |  |  |  |  |  |  | Right | .67 | | 1.08 | | .67 | | 1.08 | | 6.60 | | 16.80 | | 18.60 | | 1362.91 | |
| 11 | F | | 24 | | Chinese | | Left | 1.24 | | .85 | | 2.10 | | 2.16 | | 5.40 | | 17.10 | | 21.90 | | 1987.29 | |
|  |  |  |  |  |  |  | Right | 1.34 | | .67 | | 1.50 | | .67 | | 6.60 | | 15.30 | | 21.00 | | 1899.83 | |
| 12 | F | | 25 | | Chinese | | Left | .67 | | .85 | | 1.20 | | .95 | | 9.00 | | 18.90 | | 21.90 | | 1681.34 | |
|  |  |  |  |  |  |  | Right | 1.08 | | 1.43 | | 2.40 | | 1.50 | | 9.30 | | 17.10 | | 21.60 | | 1566.12 | |
| 13 | F | | 36 | | Chinese | | Left | .60 | | 1.50 | | 1.80 | | 1.75 | | 5.10 | | 18.30 | | 16.80 | | 1814.20 | |
|  |  |  |  |  |  |  | Right | .50 | | 1.92 | | 3.30 | | 2.58 | | 6.00 | | 17.40 | | 14.70 | | 1457.49 | |
| 14 | F | | 33 | | Chinese | | Left | 3.50 | | 1.08 | | 2.70 | | 2.58 | | 8.10 | | 19.50 | | 14.10 | | 1538.55 | |
|  |  |  |  |  |  |  | Right | 1.90 | | 1.27 | | 3.00 | | 1.92 | | 8.10 | | 20.40 | | 20.10 | | 1258.28 | |
| 15 | F | | 33 | | Chinese | | Left | 3.90 | | 1.75 | | 3.60 | | 1.75 | | 7.20 | | 20.10 | | 20.90 | | 1850.95 | |
|  |  |  |  |  |  |  | Right | 2.68 | | 1.70 | | 4.20 | | 1.92 | | 6.90 | | 18.00 | | 15.00 | | 1009.00 | |
| 16 | F | | 45 | | Chinese | | Left | .95 | | 2.28 | | 2.40 | | .85 | | 5.70 | | 19.80 | | 18.90 | | 1506.60 | |
|  |  |  |  |  |  |  | Right | .90 | | 1.34 | | 2.10 | | 2.18 | | 6.00 | | 19.50 | | 16.20 | | 1399.20 | |
| 17 | F | | 41 | | Chinese | | Left | 3.90 | | 1.27 | | 3.30 | | 2.12 | | 6.60 | | 18.30 | | 13.80 | | 1327.51 | |
|  |  |  |  |  |  |  | Right | 3.76 | | 2.68 | | 2.85 | | .67 | | 7.79 | | 13.50 | | 14.38 | | 1285.47 | |
| 18 | F | | 45 | | Chinese | | Left | 1.82 | | 1.27 | | 2.40 | | 1.70 | | 7.20 | | 19.20 | | 20.10 | | 1000.50 | |
|  |  |  |  |  |  |  | Right | .90 | | 1.50 | | 2.70 | | 1.92 | | 7.20 | | 18.00 | | 19.50 | | 1106.56 | |
| 19 | F | | 21 | | Chinese | | Left | 1.08 | | 1.34 | | 1.80 | | 1.75 | | 7.50 | | 15.90 | | 18.90 | | 1468.87 | |
|  |  |  |  |  |  |  | Right | .60 | | 1.08 | | 2.70 | | 1.75 | | 6.60 | | 17.10 | | 19.50 | | 1697.66 | |
| **Cont.Table I.2.Mesurments of the thickness of the glenoid fossa roof, anterior joint space,superior**  **joint space,posterior joint space, condylar length, condylar width,condylar height and condylar volume.** | | | | | | | | | | | | | | | | | | | | | | | |
| **No** | **Gender** | | **Age** | | **Race** | | **Side** | **Glenoid Roof Thickness** | **Condylar Position** | | | | | | **Condylar Length** | | **Condylar Width** | | **Condylar**  **height** | | **Condylar volume** | | |
|  |  |  |  |  |  |  |  |  | **AS** | | **SS** | | **PS** | |  |  |  |  |  |  |  |  |  |
| 20 | F | | 28 | | Chinese | | Left | .67 | 1.50 | | 2.40 | | 2.16 | | 5.70 | | 18.90 | | 15.00 | | 1659.94 | | |
|  |  |  |  |  |  |  | Right | .60 | 1.75 | | 2.40 | | 2.16 | | 6.90 | | 20.10 | | 14.70 | | 1364.56 | | |
| 21 | F | | 28 | | Chinese | | Left | 3.00 | 1.70 | | 3.00 | | 3.24 | | 6.30 | | 18.90 | | 25.50 | | 1475.39 | | |
|  |  |  |  |  |  |  | Right | 2.16 | 3.09 | | 3.90 | | 3.09 | | 5.10 | | 15.70 | | 21.90 | | 1267.90 | | |
| 22 | F | | 45 | | Chinese | | Left | .90 | 4.00 | | 2.40 | | 1.90 | | 7.50 | | 17.40 | | 19.20 | | 1089.20 | | |
|  |  |  |  |  |  |  | Right | .60 | 2.12 | | 3.90 | | 2.01 | | 8.40 | | 17.10 | | 21.60 | | 1801.88 | | |
| 23 | F | | 23 | | Chinese | | Left | 1.80 | 2.28 | | 2.70 | | 2.12 | | 6.00 | | 16.50 | | 16.50 | | 1178.49 | | |
|  |  |  |  |  |  |  | Right | 2.40 | 3.24 | | 2.70 | | 1.50 | | 6.60 | | 14.70 | | 14.70 | | 1286.82 | | |
| 24 | F | | 28 | | Chinese | | Left | 1.75 | 1.50 | | 3.00 | | 2.70 | | 7.80 | | 16.80 | | 13.50 | | 1947.57 | | |
|  |  |  |  |  |  |  | Right | 1.50 | 1.34 | | 2.70 | | 2.58 | | 7.80 | | 16.50 | | 15.90 | | 1775.77 | | |
| 25 | F | | 28 | | Chinese | | Left | 1.20 | .58 | | 3.90 | | 1.08 | | 9.30 | | 17.40 | | 18.30 | | 1044.17 | | |
|  |  |  |  |  |  |  | Right | .67 | 2.60 | | 3.30 | | 1.62 | | 8.40 | | 17.40 | | 19.80 | | 951.20 | | |
| 26 | M | | 43 | | Chinese | | Left | 1.34 | 1.27 | | 2.70 | | 1.70 | | 7.80 | | 21.30 | | 18.00 | | 1167.25 | | |
|  |  |  |  |  |  |  | Right | 1.20 | 1.27 | | 2.70 | | 1.90 | | 8.70 | | 21.60 | | 19.50 | | 2829.95 | | |
| 27 | M | | 34 | | Chinese | | Left | .60 | 1.92 | | 3.30 | | 1.92 | | 6.60 | | 15.60 | | 13.10 | | 2553.73 | | |
|  |  |  |  |  |  |  | Right | .95 | 1.92 | | 3.90 | | 2.42 | | 7.20 | | 16.20 | | 15.30 | | 2654.07 | | |
| 28 | M | | 33 | | Chinese | | Left | 1.20 | 1.75 | | 3.90 | | 1.70 | | 7.50 | | 21.30 | | 20.10 | | 2549.33 | | |
|  |  |  |  |  |  |  | Right | .90 | 1.50 | | 3.00 | | 2.00 | | 6.90 | | 21.00 | | 21.00 | | 2886.88 | | |
| 29 | M | | 42 | | Chinese | | Left | .80 | 1.70 | | 3.20 | | 1.40 | | 8.00 | | 16.80 | | 18.00 | | 2023.19 | | |
|  |  |  |  |  |  |  | Right | 1.20 | 2.00 | | 3.20 | | 2.20 | | 7.20 | | 17.20 | | 20.00 | | 2226.86 | | |
| 30 | M | | 18 | | Chinese | | Left | .95 | 1.92 | | 3.90 | | 2.95 | | 6.90 | | 19.50 | | 18.30 | | 1371.95 | | |
|  |  |  |  |  |  |  | Right | .60 | 2.40 | | 3.60 | | 2.16 | | 5.40 | | 21.90 | | 18.90 | | 1510.93 | | |
| 31 | M | | 44 | | Chinese | | Left | .90 | 2.50 | | 2.70 | | 1.50 | | 7.20 | | 17.10 | | 18.30 | | 1188.24 | | |
|  |  |  |  |  |  |  | Right | .90 | 2.40 | | 2.28 | | 2.12 | | 8.40 | | 15.90 | | 20.10 | | 1343.70 | | |
| 32 | M | | 44 | | Chinese | | Left | .67 | 1.75 | | 2.70 | | 1.50 | | 7.20 | | 14.70 | | 18.60 | | 1240.12 | | |
|  |  |  |  |  |  |  | Right | .95 | 2.95 | | 2.70 | | 1.08 | | 8.40 | | 15.49 | | 19.50 | | 1434.42 | | |
| 33 | M | | 18 | | Chinese | | Left | 1.24 | 1.50 | | 4.51 | | 7.26 | | 8.10 | | 14.10 | | 18.60 | | 1018.58 | | |
|  |  |  |  |  |  |  | Right | .90 | 1.50 | | 3.90 | | 7.00 | | 7.20 | | 12.90 | | 19.80 | | 1612.19 | | |
| 34 | M | | 27 | | Chinese | | Left | 1.53 | 1.75 | | 5.40 | | 4.08 | | 5.41 | | 16.20 | | 18.30 | | 1675.05 | | |
|  |  |  |  |  |  |  | Right | 1.00 | 2.28 | | 5.70 | | 3.70 | | 5.10 | | 19.50 | | 18.60 | | 1964.87 | | |
| 35 | M | | 18 | | Chinese | | Left | 1.50 | 1.30 | | 1.80 | | 1.50 | | 7.20 | | 14.70 | | 16.50 | | 1306.01 | | |
|  |  |  |  |  |  |  | Right | 2.10 | .60 | | 1.50 | | 1.75 | | 7.20 | | 15.30 | | 18.60 | | 1804.55 | | |
| 36 | M | | 21 | | Chinese | | Left | .90 | 1.93 | | 3.65 | | 3.50 | | 6.30 | | 16.20 | | 18.90 | | 1106.11 | | |
|  |  |  |  |  |  |  | Right | 1.80 | 3.23 | | 1.80 | | 1.50 | | 7.50 | | 17.70 | | 16.50 | | 1119.64 | | |
| 37 | M | | 45 | | Chinese | | Left | 1.00 | 3.80 | | 6.60 | | 6.80 | | 5.10 | | 16.80 | | 17.70 | | 1751.76 | | |
|  |  |  |  |  |  |  | Right | 1.00 | 3.24 | | 5.10 | | 5.60 | | 5.70 | | 15.60 | | 18.60 | | 2398.91 | | |
| 38 | M | | 45 | | Chinese | | Left | 1.24 | 1.34 | | 3.60 | | 2.12 | | 7.80 | | 20.00 | | 17.40 | | 1784.80 | | |
|  |  |  |  |  |  |  | Right | .90 | 2.58 | | 3.30 | | 2.16 | | 5.71 | | 21.00 | | 13.80 | | 1692.80 | | |
| **Cont.Table 1.Mesurments of the thickness of the glenoid fossa roof, anterior joint space,superior**  **joint space,posterior joint space, condylar length, condylar width,condylar height and condylar volume.** | | | | | | | | | | | | | | | | | | | | | | | |
| **No** | **Gender** | | **Age** | | **Race** | | **Side** | **Glenoid Roof Thickness** | **Condylar Position** | | | | | | **Condylar Length** | | **Condylar Width** | | **Condylar**  **height** | | | | **Condylar volume** |
|  |  |  |  |  |  |  |  |  | **AS** | | **SS** | | **PS** | |  |  |  |  |  |  |  |  |  |
| 39 | M | | 44 | | Chinese | | Left | 1.50 | 3.40 | | 3.70 | | 1.50 | | 6.90 | | 17.70 | | 23.10 | | | | 1691.71 |
|  |  |  |  |  |  |  | Right | 1.90 | 2.28 | | 4.80 | | 4.18 | | 8.40 | | 15.00 | | 24.30 | | | | 1505.06 |
| 40 | M | | 41 | | Chinese | | Left | .90 | 1.50 | | 1.50 | | .60 | | 8.40 | | 21.30 | | 15.00 | | | | 1757.29 |
|  |  |  |  |  |  |  | Right | .90 | 1.50 | | 2.10 | | 1.30 | | 7.50 | | 21.90 | | 17.40 | | | | 2046.37 |
| 41 | M | | 27 | | Chinese | | Left | 1.50 | 1.30 | | 2.70 | | 2.12 | | 8.40 | | 16.50 | | 23.30 | | | | 1062.19 |
|  |  |  |  |  |  |  | Right | 1.90 | 2.00 | | 3.30 | | 2.70 | | 8.10 | | 16.50 | | 24.30 | | | | 1075.96 |
| 42 | M | | 27 | | Chinese | | Left | 1.80 | .60 | | 1.50 | | 2.50 | | 8.40 | | 18.00 | | 18.90 | | | | 1260.98 |
|  |  |  |  |  |  |  | Right | .90 | 1.80 | | 1.80 | | 3.19 | | 8.50 | | 15.30 | | 20.33 | | | | 1779.41 |
| 43 | M | | 43 | | Chinese | | Left | 1.50 | .90 | | 2.70 | | 2.30 | | 6.90 | | 18.90 | | 17.70 | | | | 1462.24 |
|  |  |  |  |  |  |  | Right | 1.00 | 2.68 | | 2.70 | | 2.12 | | 5.40 | | 19.50 | | 16.20 | | | | 1442.25 |
| 44 | M | | 44 | | Chinese | | Left | 1.00 | 3.00 | | 6.00 | | 4.00 | | 6.30 | | 19.80 | | 16.20 | | | | 1918.72 |
|  |  |  |  |  |  |  | Right | 2.10 | 1.90 | | 7.80 | | 5.40 | | 8.10 | | 16.80 | | 13.20 | | | | 1806.96 |
| 45 | M | | 40 | | Chinese | | Left | 1.80 | 2.10 | | 6.00 | | 4.17 | | 7.80 | | 21.00 | | 22.80 | | | | 1139.56 |
|  |  |  |  |  |  |  | Right | 1.00 | 1.90 | | 4.20 | | 4.20 | | 9.00 | | 17.70 | | 21.00 | | | | 1142.20 |
| 46 | M | | 31 | | Chinese | | Left | 1.00 | 1.70 | | 6.60 | | 4.50 | | 8.10 | | 14.70 | | 20.40 | | | | 1642.89 |
|  |  |  |  |  |  |  | Right | 2.16 | 1.50 | | 7.00 | | 6.00 | | 9.50 | | 17.70 | | 17.40 | | | | 1396.82 |
| 47 | M | | 42 | | Chinese | | Left | 3.00 | .90 | | 3.00 | | 1.50 | | 6.60 | | 20.10 | | 17.40 | | | | 1401.37 |
|  |  |  |  |  |  |  | Right | 1.50 | 2.18 | | 3.00 | | 1.30 | | 6.90 | | 17.40 | | 17.70 | | | | 2084.47 |
| 48 | M | | 27 | | Chinese | | Left | 2.12 | 1.70 | | 2.40 | | 3.19 | | 8.10 | | 18.00 | | 22.80 | | | | 1562.20 |
|  |  |  |  |  |  |  | Right | 1.00 | 1.50 | | 4.20 | | 3.00 | | 8.10 | | 20.40 | | 23.40 | | | | 1765.72 |
| 49 | M | | 41 | | Chinese | | Left | 1.00 | 2.60 | | 3.30 | | 1.80 | | 7.80 | | 17.40 | | 23.10 | | | | 1691.71 |
|  |  |  |  |  |  |  | Right | 1.00 | .00 | | 4.50 | | 5.30 | | 8.40 | | 16.50 | | 24.60 | | | | 1505.06 |
| 50 | M | | 34 | | Chinese | | Left | 1.60 | 2.60 | | 4.00 | | 1.50 | | 5.60 | | 18.80 | | 22.00 | | | | 1126.82 |
|  |  |  |  |  |  |  | Right | 1.40 | 2.20 | | 4.00 | | 2.04 | | 6.40 | | 19.60 | | 18.80 | | | | 1289.96 |
| 51 | F | | 27 | | Malay | | Left | .90 | 1.62 | | 3.30 | | 2.58 | | 6.60 | | 12.90 | | 12.01 | | | | 1951.68 |
|  |  |  |  |  |  |  | Right | .90 | 2.12 | | 1.50 | | .76 | | 5.70 | | 17.10 | | 15.60 | | | | 1677.15 |
| 52 | F | | 41 | | Malay | | Left | 1.62 | 1.70 | | 2.40 | | 2.58 | | 7.20 | | 17.70 | | 16.80 | | | | 1691.09 |
|  |  |  |  |  |  |  | Right | 3.00 | 1.08 | | 3.30 | | 2.68 | | 7.50 | | 19.50 | | 17.40 | | | | 1583.31 |
| 53 | F | | 26 | | Malay | | Left | 1.92 | 1.75 | | 3.60 | | 2.97 | | 5.40 | | 12.30 | | 12.00 | | | | 854.52 |
|  |  |  |  |  |  |  | Right | 5.14 | 1.92 | | 4.20 | | 2.28 | | 6.60 | | 10.30 | | 12.60 | | | | 874.17 |
| 54 | F | | 23 | | Malay | | Left | 1.80 | 1.70 | | 2.40 | | 2.16 | | 8.10 | | 18.00 | | 27.60 | | | | 2538.67 |
|  |  |  |  |  |  |  | Right | 1.24 | 1.08 | | 2.40 | | 2.55 | | 7.20 | | 17.10 | | 22.20 | | | | 2647.70 |
| 55 | F | | 32 | | Malay | | Left | .90 | 1.34 | | 1.80 | | 1.80 | | 6.30 | | 20.10 | | 19.20 | | | | 1478.38 |
|  |  |  |  |  |  |  | Right | .67 | 1.92 | | 1.50 | | 1.08 | | 6.30 | | 19.20 | | 12.60 | | | | 1150.42 |
| 56 | F | | 21 | | Malay | | Left | .90 | 1.50 | | 1.50 | | 2.34 | | 6.60 | | 15.60 | | 10.80 | | | | 890.29 |
|  |  |  |  |  |  |  | Right | .60 | 1.24 | | 1.56 | | 1.70 | | 6.60 | | 15.00 | | 10.80 | | | | 868.50 |
| 57 | F | | 44 | | Malay | | Left | .90 | 1.50 | | .90 | | .90 | | 9.00 | | 15.30 | | 17.40 | | | | 982.75 |
|  |  |  |  |  |  |  | Right | .90 | 1.50 | | 3.60 | | 3.60 | | 8.70 | | 15.30 | | 15.30 | | | | 1056.77 |
| **Cont.Table 1.Mesurments of the thickness of the glenoid fossa roof, anterior joint space,superior**  **joint space,posterior joint space, condylar length, condylar width,condylar height and condylar volume.** | | | | | | | | | | | | | | | | | | | | | | | |
| **No** | | **Gender** | | **Age** | | **Race** | **Side** | **Glenoid Roof Thickness** | **Condylar Position** | | | | | | **Condylar Length** | | **Condylar Width** | | **Condylar**  **height** | | | | **Condylar volume** |
|  |  |  |  |  |  |  |  |  | **AS** | | **SS** | | **PS** | |  |  |  |  |  |  |  |  |  |
| 58 | | F | | 30 | | Malay | Left | .60 | 1.75 | | 3.30 | | 3.30 | | 6.60 | | 13.50 | | 19.50 | | | | 1493.66 |
|  |  |  |  |  |  |  | Right | .90 | 1.62 | | 1.80 | | 2.58 | | 7.20 | | 14.40 | | 20.40 | | | | 1784.53 |
| 59 | | F | | 39 | | Malay | Left | 1.20 | 1.50 | | 2.40 | | 2.50 | | 6.60 | | 16.50 | | 25.50 | | | | 1452.54 |
|  |  |  |  |  |  |  | Right | .60 | 2.30 | | 3.30 | | 1.50 | | 9.00 | | 17.40 | | 18.00 | | | | 1453.45 |
| 60 | | F | | 31 | | Malay | Left | 1.50 | 1.27 | | 1.20 | | .90 | | 8.40 | | 20.40 | | 21.90 | | | | 1318.44 |
|  |  |  |  |  |  |  | Right | 1.80 | 1.20 | | 2.10 | | 1.20 | | 8.20 | | 19.80 | | 22.50 | | | | 1463.89 |
| 61 | | F | | 19 | | Malay | Left | 1.20 | 2.00 | | 2.10 | | 1.60 | | 7.22 | | 16.50 | | 20.40 | | | | 1177.18 |
|  |  |  |  |  |  |  | Right | 1.80 | 1.20 | | 2.40 | | 2.00 | | 7.50 | | 15.60 | | 15.60 | | | | 1156.61 |
| 62 | | F | | 21 | | Malay | Left | 1.08 | 1.30 | | 2.70 | | 2.16 | | 7.50 | | 15.60 | | 14.40 | | | | 1426.17 |
|  |  |  |  |  |  |  | Right | .90 | 1.20 | | 2.70 | | 1.50 | | 7.50 | | 17.10 | | 17.70 | | | | 1504.84 |
| 63 | | F | | 30 | | Malay | Left | .90 | 1.34 | | 3.00 | | 2.18 | | 5.40 | | 16.50 | | 17.70 | | | | 1020.67 |
|  |  |  |  |  |  |  | Right | 1.24 | 1.60 | | 2.40 | | 1.24 | | 7.50 | | 16.80 | | 18.90 | | | | 1362.64 |
| 64 | | F | | 24 | | Malay | Left | 1.24 | .90 | | 2.10 | | 2.40 | | 7.80 | | 14.40 | | 16.20 | | | | 1021.72 |
|  |  |  |  |  |  |  | Right | 1.20 | 1.08 | | 1.80 | | 1.50 | | 8.40 | | 17.70 | | 15.90 | | | | 1080.80 |
| 65 | | F | | 28 | | Malay | Left | 1.34 | 1.27 | | 3.30 | | 2.16 | | 9.00 | | 17.40 | | 18.00 | | | | 1229.06 |
|  |  |  |  |  |  |  | Right | 1.23 | 1.20 | | 3.00 | | 2.20 | | 8.50 | | 17.00 | | 17.50 | | | | 520.92 |
| 66 | | F | | 36 | | Malay | Left | 3.00 | 2.12 | | 4.00 | | 1.92 | | 7.80 | | 17.10 | | 17.00 | | | | 982.03 |
|  |  |  |  |  |  |  | Right | 3.00 | 2.60 | | 3.90 | | 3.30 | | 6.60 | | 17.10 | | 15.00 | | | | 875.87 |
| 67 | | F | | 21 | | Malay | Left | .90 | 1.50 | | 2.70 | | 1.27 | | 7.50 | | 17.10 | | 18.50 | | | | 1482.03 |
|  |  |  |  |  |  |  | Right | 1.24 | .90 | | 2.40 | | 1.20 | | 7.80 | | 17.10 | | 17.10 | | | | 1679.81 |
| 68 | | F | | 25 | | Malay | Left | 1.50 | 2.16 | | 2.70 | | 1.70 | | 6.60 | | 16.50 | | 19.20 | | | | 1591.24 |
|  |  |  |  |  |  |  | Right | .90 | 1.50 | | 3.30 | | 2.50 | | 7.80 | | 16.20 | | 17.40 | | | | 1513.94 |
| 69 | | F | | 30 | | Malay | Left | 1.50 | 1.34 | | 1.50 | | 1.27 | | 7.50 | | 18.30 | | 17.10 | | | | 968.16 |
|  |  |  |  |  |  |  | Right | .90 | 2.30 | | 1.50 | | 1.70 | | 7.50 | | 13.50 | | 15.30 | | | | 815.15 |
| 70 | | F | | 27 | | Malay | Left | .90 | .90 | | 1.50 | | 1.20 | | 9.00 | | 15.00 | | 19.80 | | | | 1151.84 |
|  |  |  |  |  |  |  | Right | .90 | 1.20 | | 1.50 | | .60 | | 9.00 | | 15.00 | | 16.80 | | | | 1127.04 |
| 71 | | F | | 18 | | Malay | Left | 1.80 | 1.60 | | 3.50 | | 3.70 | | 8.00 | | 15.00 | | 18.00 | | | | 1507.15 |
|  |  |  |  |  |  |  | Right | 1.03 | 2.20 | | 3.30 | | 3.50 | | 7.50 | | 15.00 | | 16.25 | | | | 1351.79 |
| 72 | | F | | 26 | | Malay | Left | 2.50 | 1.30 | | 2.16 | | 2.28 | | 6.00 | | 17.70 | | 12.90 | | | | 2568.47 |
|  |  |  |  |  |  |  | Right | 1.50 | .90 | | 4.80 | | 5.80 | | 6.90 | | 17.70 | | 13.20 | | | | 2461.19 |
| 73 | | F | | 23 | | Malay | Left | 1.20 | 2.80 | | 2.70 | | 1.27 | | 5.70 | | 16.50 | | 12.60 | | | | 995.32 |
|  |  |  |  |  |  |  | Right | .90 | 1.92 | | 1.80 | | 1.90 | | 7.50 | | 13.30 | | 13.80 | | | | 1040.67 |
| 74 | | F | | 25 | | Malay | Left | 1.20 | 2.60 | | 2.70 | | 2.00 | | 6.90 | | 16.00 | | 12.90 | | | | 1112.16 |
|  |  |  |  |  |  |  | Right | 1.20 | 2.00 | | 2.40 | | 1.90 | | 6.00 | | 16.00 | | 15.00 | | | | 1145.22 |
| 75 | | F | | 35 | | Malay | Left | 2.00 | 1.25 | | 3.00 | | 2.24 | | 6.50 | | 18.00 | | 14.25 | | | | 1070.81 |
|  |  |  |  |  |  |  | Right | 1.25 | 1.40 | | 3.00 | | 1.80 | | 6.70 | | 16.25 | | 14.25 | | | | 1091.45 |
| 76 | | M | | 22 | | Malay | Left | 1.20 | 1.70 | | 3.20 | | 1.13 | | 7.70 | | 18.00 | | 24.30 | | | | 1763.20 |
|  |  |  |  |  |  |  | Right | .90 | 2.00 | | 3.20 | | 1.70 | | 9.20 | | 13.60 | | 18.00 | | | | 2406.80 |
|  | | | | | | | | | | | | | | | | | | | | | | | |
| **Cont.Table I.2.Mesurments of the thickness of the glenoid fossa roof, anterior joint space, superior joint space, posterior joint space, condylar length, condylar width, condylar height and condylar volume.** | | | | | | | | | | | | | | | | | | | | | | | |
| **No** | | **Gender** | | **Age** | | **Race** | **Side** | **Glenoid Roof Thickness** | **Condylar Position** | | | | | | **Condylar Length** | | **Condylar Width** | | **Condylar**  **height** | | | | **Condylar volume** |
|  |  |  |  |  |  |  |  |  | **AS** | | **SS** | | **PS** | |  |  |  |  |  |  |  |  |  |
| 77 | | M | | 20 | | Malay | Left | 2.10 | 1.50 | | 3.00 | | 2.00 | | 7.50 | | 20.70 | | 17.10 | | | | 1531.22 |
|  |  |  |  |  |  |  | Right | 2.77 | 1.34 | | 2.70 | | 2.55 | | 7.50 | | 21.00 | | 16.50 | | | | 2711.52 |
| 78 | | M | | 25 | | Malay | Left | 1.79 | 1.77 | | 2.70 | | 1.44 | | 7.50 | | 20.00 | | 15.00 | | | | 2528.04 |
|  |  |  |  |  |  |  | Right | 2.40 | 3.60 | | 3.60 | | 1.40 | | 6.80 | | 17.20 | | 12.80 | | | | 2159.91 |
| 79 | | M | | 25 | | Malay | Left | .90 | 2.50 | | 3.00 | | 1.50 | | 5.40 | | 16.20 | | 18.30 | | | | 2304.38 |
|  |  |  |  |  |  |  | Right | .95 | 1.90 | | 1.50 | | 1.30 | | 6.60 | | 17.40 | | 16.50 | | | | 2126.12 |
| 80 | | M | | 25 | | Malay | Left | 2.70 | 2.20 | | 3.90 | | 2.70 | | 6.90 | | 19.20 | | 19.80 | | | | 1530.57 |
|  |  |  |  |  |  |  | Right | 2.77 | 4.00 | | 3.50 | | 2.16 | | 7.20 | | 19.20 | | 20.40 | | | | 2357.56 |
| 81 | | M | | 18 | | Malay | Left | 1.08 | 1.08 | | 4.50 | | 2.34 | | 6.30 | | 18.90 | | 20.10 | | | | 1274.33 |
|  |  |  |  |  |  |  | Right | .90 | 2.40 | | 3.60 | | 2.16 | | 8.10 | | 18.90 | | 19.80 | | | | 1281.05 |
| 82 | | M | | 22 | | Malay | Left | 1.90 | 1.70 | | 5.00 | | 3.00 | | 6.30 | | 19.80 | | 15.00 | | | | 1224.03 |
|  |  |  |  |  |  |  | Right | .90 | 1.08 | | 4.50 | | 2.30 | | 8.00 | | 17.10 | | 15.60 | | | | 1519.01 |
| 83 | | M | | 45 | | Malay | Left | 3.00 | 1.50 | | 2.12 | | 2.01 | | 6.90 | | 18.60 | | 14.70 | | | | 1747.00 |
|  |  |  |  |  |  |  | Right | 2.20 | 1.08 | | 4.00 | | 5.00 | | 7.50 | | 19.50 | | 14.00 | | | | 1615.55 |
| 84 | | M | | 30 | | Malay | Left | 1.50 | 1.70 | | 3.90 | | 4.20 | | 6.30 | | 14.40 | | 15.90 | | | | 1866.32 |
|  |  |  |  |  |  |  | Right | .90 | 2.12 | | 3.30 | | 3.70 | | 6.30 | | 13.50 | | 15.50 | | | | 1682.92 |
| 85 | | M | | 19 | | Malay | Left | 1.50 | .60 | | .90 | | .80 | | 7.80 | | 15.60 | | 19.50 | | | | 1184.66 |
|  |  |  |  |  |  |  | Right | .60 | 1.80 | | 2.30 | | 1.50 | | 6.60 | | 13.20 | | 17.40 | | | | 1034.29 |
| 86 | | M | | 23 | | Malay | Left | 1.20 | 1.44 | | 2.80 | | 1.60 | | 7.60 | | 21.60 | | 20.40 | | | | 1800.11 |
|  |  |  |  |  |  |  | Right | 1.20 | 1.60 | | 3.60 | | 2.80 | | 8.40 | | 18.40 | | 20.00 | | | | 1867.45 |
| 87 | | M | | 45 | | Malay | Left | 1.80 | 1.80 | | 3.30 | | 2.16 | | 5.70 | | 17.70 | | 19.80 | | | | 3156.15 |
|  |  |  |  |  |  |  | Right | .90 | 1.90 | | 3.60 | | 2.90 | | 7.50 | | 17.40 | | 19.50 | | | | 3576.57 |
| 88 | | M | | 36 | | Malay | Left | 2.70 | 2.12 | | 3.90 | | 1.50 | | 6.60 | | 20.70 | | 18.00 | | | | 866.64 |
|  |  |  |  |  |  |  | Right | 1.90 | 2.40 | | 3.00 | | .90 | | 5.40 | | 18.70 | | 18.00 | | | | 1330.59 |
| 89 | | M | | 28 | | Malay | Left | 1.50 | 1.50 | | 3.90 | | 2.40 | | 8.10 | | 15.30 | | 15.30 | | | | 1078.30 |
|  |  |  |  |  |  |  | Right | 1.20 | 1.20 | | 3.00 | | 2.28 | | 7.20 | | 18.00 | | 15.90 | | | | 1238.21 |
| 90 | | M | | 24 | | Malay | Left | .90 | 2.20 | | 3.60 | | 3.50 | | 5.70 | | 20.70 | | 14.70 | | | | 1039.41 |
|  |  |  |  |  |  |  | Right | .90 | 2.80 | | 4.50 | | 3.60 | | 6.30 | | 18.30 | | 14.70 | | | | 883.56 |
| 91 | | M | | 24 | | Malay | Left | 1.50 | 2.12 | | 6.00 | | 4.00 | | 9.00 | | 17.40 | | 20.40 | | | | 1927.50 |
|  |  |  |  |  |  |  | Right | 1.80 | 2.50 | | 3.30 | | 2.12 | | 8.70 | | 17.20 | | 20.70 | | | | 2002.09 |
| 92 | | M | | 29 | | Malay | Left | 2.40 | 2.00 | | 3.00 | | 1.50 | | 7.80 | | 17.20 | | 19.80 | | | | 2115.61 |
|  |  |  |  |  |  |  | Right | 3.00 | 1.50 | | 3.70 | | 2.30 | | 6.90 | | 17.00 | | 18.30 | | | | 2170.20 |
| 93 | | M | | 34 | | Malay | Left | .90 | 1.08 | | 1.30 | | 1.50 | | 6.60 | | 18.60 | | 18.00 | | | | 1143.08 |
|  |  |  |  |  |  |  | Right | .60 | 1.50 | | 1.80 | | .60 | | 8.10 | | 16.80 | | 16.80 | | | | 1151.78 |
| 94 | | M | | 35 | | Malay | Left | 2.16 | 1.90 | | 3.30 | | 1.08 | | 6.60 | | 19.50 | | 16.80 | | | | 1846.89 |
|  |  |  |  |  |  |  | Right | .60 | 1.50 | | 3.60 | | 2.00 | | 7.80 | | 21.00 | | 18.00 | | | | 2094.87 |
| 95 | | M | | 24 | | Malay | Left | 3.00 | 1.60 | | 3.00 | | 2.20 | | 9.30 | | 14.20 | | 11.40 | | | | 836.43 |
|  |  |  |  |  |  |  | Right | 3.00 | 1.00 | | 2.10 | | 3.00 | | 8.70 | | 13.80 | | 10.80 | | | | 702.09 |
| **Cont.Table 1.Mesurments of the thickness of the glenoid fossa roof, anterior joint space, superior joint space, posterior joint space, condylar length, condylar width, condylar height and condylar volume.** | | | | | | | | | | | | | | | | | | | | | | | |
| **No** | | **Gender** | | **Age** | | **Race** | **Side** | **Glenoid Roof Thickness** | **Condylar Position** | | | | | | **Condylar Length** | | **Condylar Width** | | **Condylar**  **height** | | | | **Condylar volume** |
|  |  |  |  |  |  |  |  |  | **AS** | | **SS** | | **PS** | |  |  |  |  |  |  |  |  |  |
| 96 | | M | | 33 | | Malay | Left | 2.70 | 1.30 | | 3.30 | | 1.90 | | 7.50 | | 16.00 | | 17.70 | | | | 1411.06 |
|  |  |  |  |  |  |  | Right | 3.00 | 2.40 | | 4.00 | | 2.00 | | 7.80 | | 16.20 | | 18.90 | | | | 2118.56 |
| 97 | | M | | 32 | | Malay | Left | .95 | 1.60 | | 4.00 | | 1.80 | | 6.30 | | 22.50 | | 19.80 | | | | 1578.11 |
|  |  |  |  |  |  |  | Right | .60 | 1.08 | | 3.00 | | 2.50 | | 7.80 | | 21.00 | | 22.50 | | | | 1665.50 |
| 98 | | M | | 27 | | Malay | Left | 1.20 | 1.08 | | 3.30 | | 3.09 | | 8.40 | | 15.90 | | 15.30 | | | | 1589.06 |
|  |  |  |  |  |  |  | Right | .90 | 2.30 | | 2.70 | | 2.40 | | 7.20 | | 13.50 | | 15.90 | | | | 1198.86 |
| 99 | | M | | 21 | | Malay | Left | 1.50 | 1.30 | | 2.70 | | 1.70 | | 6.90 | | 25.00 | | 14.10 | | | | 1864.93 |
|  |  |  |  |  |  |  | Right | .90 | 2.18 | | 3.70 | | 2.70 | | 6.60 | | 21.60 | | 16.50 | | | | 1972.92 |
| 100 | | M | | 21 | | Malay | Left | .90 | 2.18 | | 1.50 | | 1.08 | | 7.50 | | 19.80 | | 14.70 | | | | 1022.10 |
|  |  |  |  |  |  |  | Right | .90 | .70 | | 3.50 | | 2.50 | | 7.50 | | 18.90 | | 12.90 | | | | 1327.95 |
