# Supplementary material for: Assessment of Condyle and Glenoid Fossa Morphology Using CBCT in South-East Asians
Source: PLoS One. 2015 Mar 24;10(3):e0121682. doi: 10.1371/journal.pone.0121682 (PMC4372412; doi:10.1371/journal.pone.0121682)
Supplement: S2 Table — (DOCX) [file pone.0121682.s002.docx]

**Supporting Information**

**(S2)**

**Table 2. Measurement of the condyle and glenoid fossa morphology for the reliability test I (Intraclass correlation).**

| **Variable** | **No.** | **Original reading** | **Second reading** | **Third reading** |
| --- | --- | --- | --- | --- |
| **Glenoid roof thickness** | 1 | 2.01 | 2.6 | 2 |
|  | 2 | 1.7 | 1.3 | 1.5 |
|  | 3 | 1.6 | 1.5 | 1.7 |
|  | 4 | 1.24 | 3.65 | 1.5 |
|  | 5 | 0.76 | 0.9 | 2 |
|  | 6 | 0.9 | 0.9 | 1.5 |
|  | 7 | 0.88 | 0.8 | 1.2 |
|  | 8 | 0.6 | 0.5 | 2 |
|  | 9 | 0.9 | 0.7 | 1.5 |
|  | 10 | 0.7 | 0.5 | 0.9 |
|  | 11 | 0.9 | 0.8 | 0.9 |
|  | 12 | 0.9 | 0.8 | 0.9 |
|  | 13 | 0.9 | 1 | 1.5 |
|  | 14 | 1.5 | 1 | 1.5 |
|  | 15 | 0.6 | 1.5 | 1.5 |
|  | 16 | 0.6 | 0.9 | 1 |
|  | 17 | 0.9 | 0.9 | 1 |
|  | 18 | 0.6 | 0.9 | 1 |
|  | 19 | 0.67 | 0.76 | 0.7 |
|  | 20 | 0.67 | 0.78 | 0.9 |
| **Anterior space** | 1 | 2.42 | 2 | 2.96 |
|  | 2 | 2.5 | 2.7 | 2.6 |
|  | 3 | 1.5 | 1 | 0.9 |
|  | 4 | 2.4 | 2.8 | 2.6 |
|  | 5 | 2.01 | 2.3 | 2 |
|  | 6 | 1.9 | 2 | 1.8 |
|  | 7 | 1.4 | 1.6 | 1.5 |
|  | 8 | 0.6 | 0.9 | 1.2 |
|  | 9 | 1.7 | 1 | 1.5 |
|  | 10 | 1.6 | 1.5 | 1.3 |
|  | 11 | 2.4 | 2 | 2.2 |
|  | 12 | 0.85 | 0.7 | 0.9 |
|  | 13 | 1.5 | 1 | 1.6 |
|  | 14 | 2.16 | 2.01 | 2.54 |
|  | 15 | 0.95 | 0.9 | 1.30 |
|  | 16 | 1.2 | 1. | 1.3 |
|  | 17 | 1.7 | 1.5 | 1.5 |
|  | 18 | 1.2 | 1.5 | 1 |
|  | 19 | 1.08 | 1.09 | 1.2 |
|  | 20 | 1.08 | 1 | 1.5 |

**Cont. Table 2. Measurement of the condyle and glenoid fossa morphology for the reliability test ( (Intraclass correlation)**

| **Variable** | **No.** | **Original reading** | **Second reading** | **Third reading** |
| --- | --- | --- | --- | --- |
| **Superior space** | 1 | 6.75 | 6.50 | 6.00 |
|  | 2 | 5.51 | 5 | 5.20 |
|  | 3 | 2.1 | 2.2 | 2.4 |
|  | 4 | 2 | 2 | 2.6 |
|  | 5 | 2.1 | 2.2 | 2.7 |
|  | 6 | 2.1 | 2.74 | 2.56 |
|  | 7 | 1.5 | 1.32 | 1.54 |
|  | 8 | 2.1 | 2.2 | 2.1 |
|  | 9 | 2.1 | 2.5 | 2.45 |
|  | 10 | 2.1 | 2.2 | 2.4 |
|  | 11 | 1.8 | 1 | 1.5 |
|  | 12 | 2.4 | 2.1 | 2.3 |
|  | 13 | 1.2 | 1.5 | 1.3 |
|  | 14 | 1.2 | 1.4 | 1.5 |
|  | 15 | 0.9 | 0.9 | 1 |
|  | 16 | 1.27 | 1.20 | 1.1 |
|  | 17 | 2.1 | 2.2 | 2 |
|  | 18 | 2.1 | 2.1 | 2.54 |
|  | 19 | 1.34 | 1.32 | 1.54 |
|  | 20 | 0.67 | 0.90 | 1.67 |
| **Posterior space** | 1 | 6 | 6.1 | 6.3 |
|  | 2 | 5.9 | 5.5 | 5 |
|  | 3 | 2.66 | 2.4 | 2.6 |
|  | 4 | 2.5 | 2.3 | 2.2 |
|  | 5 | 2.16 | 2.2 | 2.3 |
|  | 6 | 2.16 | 2.9 | 2.15 |
|  | 7 | 1.5 | 1.7 | 1.5 |
|  | 8 | 1.88 | 1 | 1.5 |
|  | 9 | 1.7 | 1.4 | 1.5 |
|  | 10 | 1.5 | 1.5 | 1.3 |
|  | 11 | 1.72 | 1.34 | 1.44 |
|  | 12 | 1.7 | 1.45 | 1.32 |
|  | 13 | 0.5 | 1 | 0.9 |
|  | 14 | 1.08 | 1.06 | 1 |
|  | 15 | 1.5 | 1.9 | 1.7 |
|  | 16 | 1.27 | 1.45 | 1.35 |
|  | 17 | 1.53 | 1.84 | 1.34 |
|  | 18 | 1.24 | 1.2 | 1.5 |
|  | 19 | 1.08 | 1.1 | 1.09 |
|  | 20 | 1.08 | 1.3 | 1.6 |

**Cont. Table 2. Measurement of the condyle and glenoid fossa morphology for the reliability test**

**( (Intraclass correlation)**

| **Variable** | **No.** | **Original reading** | **Second reading** | **Third reading** |
| --- | --- | --- | --- | --- |
| **Condylar length** | 1 | 6.52 | 5 | 5.5 |
|  | 2 | 6.63 | 6. | 5.5 |
|  | 3 | 6.7 | 6.70 | 6.4 |
|  | 4 | 5.5 | 4.5 | 5.9 |
|  | 5 | 5.7 | 4.7 | 3.9 |
|  | 6 | 5.7 | 4.9 | 3.4 |
|  | 7 | 5.2 | 5 | 4 |
|  | 8 | 6.93 | 7 | 6.5 |
|  | 9 | 5.88 | 6 | 5 |
|  | 10 | 6 | 6 | 5.23 |
|  | 11 | 7.8 | 7.24 | 7 |
|  | 12 | 8.4 | 8 | 6 |
|  | 13 | 7.2 | 6.43 | 6.45 |
|  | 14 | 6.9 | 6 | 5.89 |
|  | 15 | 6.6 | 6.98 | 6 |
|  | 16 | 7.2 | 7.98 | 6.90 |
|  | 17 | 7.8 | 7.4 | 7.9 |
|  | 18 | 8.1 | 8.6 | 7.84 |
|  | 19 | 7.13 | 7 | 6.89 |
|  | 20 | 6.6 | 6.11 | 7.27 |
| **Condylar width** | 1 | 18.62 | 18 | 18.48 |
|  | 2 | 18.91 | 18.95 | 18,57 |
|  | 3 | 21.6 | 20.10 | 20.78 |
|  | 4 | 22.8 | 21.89 | 21.13 |
|  | 5 | 22.4 | 23.87 | 21.98 |
|  | 6 | 22.5 | 20.96 | 20 |
|  | 7 | 19.2 | 20.98 | 20.2 |
|  | 8 | 18.3 | 19.56 | 18.4 |
|  | 9 | 15.9 | 15.78 | 16 |
|  | 10 | 15.91 | 15.8 | 16 |
|  | 11 | 21.6 | 21.7 | 20.89 |
|  | 12 | 18.3 | 19.8 | 19.5 |
|  | 13 | 11.1 | 11.98 | 11.5 |
|  | 14 | 12.3 | 12.12 | 11 |
|  | 15 | 13.2 | 13.78 | 13.89 |
|  | 16 | 15 | 15.98 | 15.44 |
|  | 17 | 18.9 | 18.1 | 18.5 |
|  | 18 | 18.3 | 19 | 18.44 |
|  | 19 | 15.6 | 15 | 14.78 |
|  | 20 | 16.8 | 16.54 | 17 |

**Cont. Table 2. Measurement of the condyle and glenoid fossa morphology for the reliability test K (Intraclass correlation)**

| **Variable** | **No.** | **Original reading** | **Second reading** | **Third reading** |
| --- | --- | --- | --- | --- |
| **Condylar height** | 1 | 18.3 | 18.45 | 17.45 |
|  | 2 | 17.7 | 17.5 | 18,65 |
|  | 3 | 17.6 | 17.56 | 17.89 |
|  | 4 | 17.86 | 17.94 | 18 |
|  | 5 | 15.3 | 15 | 14.56 |
|  | 6 | 15.02 | 16 | 16.4 |
|  | 7 | 15 | 14 | 15.87 |
|  | 8 | 13.2 | 13 | 13.8 |
|  | 9 | 15 | 16 | 15.89 |
|  | 10 | 14.7 | 14.8 | 14.9 |
|  | 11 | 14.4 | 13.65 | 13 |
|  | 12 | 15 | 13 | 14.98 |
|  | 13 | 23.4 | 22.85 | 21 |
|  | 14 | 21.1 | 21.9 | 22.3 |
|  | 15 | 16.8 | 16.8 | 16 |
|  | 16 | 17.1 | 17.78 | 17.98 |
|  | 17 | 16.8 | 16.9 | 16.87 |
|  | 18 | 16.5 | 15 | 15.4 |
|  | 19 | 18.6 | 17.5 | 18.9 |
|  | 20 | 18.6 | 18 | 18.9 |
| **Condylar volume** | 1 | 1255 | 1445 | 1345.6 |
|  | 2 | 1821.36 | 1634.67 | 1743.45 |
|  | 3 | 1182.85 | 1445.67 | 1335.56 |
|  | 4 | 1248.62 | 1334.77 | 1445.7 |
|  | 5 | 1322.69 | 1624.56 | 1554.5 |
|  | 6 | 1035.53 | 1633.45 | 1556.9 |
|  | 7 | 1192.6 | 1485.9 | 1395.57 |
|  | 8 | 1250.71 | 1235.65 | 1238.58 |
|  | 9 | 1751.49 | 1835.59 | 1938.78 |
|  | 10 | 1875.8 | 1738.56 | 1839.78 |
|  | 11 | 1069.35 | 1356.49 | 1773.67 |
|  | 12 | 1236.63 | 1356.78 | 1246.89 |
|  | 13 | 1007.34 | 1045.67 | 1073.67 |
|  | 14 | 1412.89 | 1468.59 | 1365.9 |
|  | 15 | 1701.57 | 1656.98 | 1709.7 |
|  | 16 | 1585.16 | 1593.34 | 1468.67 |
|  | 17 | 1449.25 | 1375.59 | 1456.78 |
|  | 18 | 1440.67 | 1795.98 | 1598.67 |
|  | 19 | 1320.92 | 1875.78 | 1997.5 |
|  | 20 | 1362.91 | 1698.90 | 1757.7 |
